# POTENTIAL CLINICAL CONSEQUENCES OF ADMINISTRATIVE ISSUES REGARDING MEDICATION IN HOME CARE PATIENTS

**DOI:** 10.1093/geroni/igz038.180

**Published:** 2019-11-08

**Authors:** Nienke E Dijkstra, Carolien Sino, Marieke J Schuurmans, Marcel L Bouvy, Aline Bouwes, Rob R Heerdink

**Affiliations:** 1 University of Applied Sciences Utrecht, Utrecht, Netherlands; 2 University of Applied Sciences Utrecht, Utrecht, Utrecht, Netherlands; 3 Education Center, UMC Utrecht Academy, University Medical Center Utrecht, Utrecht, Utrecht, Netherlands

## Abstract

Home care professionals observe drug-related problems (DRPs) as administrative problems (e.g. inconsistent registration of (changes in) drug prescription) and side effects which may have clinical consequences for older patients. This study aims to determine the potential clinical impact of administrative problems. A retrospective descriptive study was performed, using reports of home care professionals of the eHOME system (system that assist monitoring/reporting DRPs). Administrative problems of a one year period were assessed by three experts on potential discomfort/clinical deterioration using a 3-point scale. 309 DRPs of 120 out of 451 patients (age ≥65) were assessed. Problems involved undelivered medication administration record lists (n=103,33.3%), inconsistent registration of drug prescription (n=188,60.9%) and insufficient drug delivery (n=18,5.8%). 58.2% of the DRPs had the potential to cause moderate to severe discomfort or clinical deterioration. The results underlines the importance of the observation function of home care professionals and the need to improve pharmaceutical administration issues.

